# An induced rebinding model of antigen discrimination^☆^

**DOI:** 10.1016/j.it.2014.02.002

**Published:** 2014-04

**Authors:** Omer Dushek, P. Anton van der Merwe

**Affiliations:** 1 Sir William Dunn School of Pathology, University of Oxford, Oxford, UK; 2 Wolfson Centre for Mathematical Biology, University of Oxford, Oxford, UK

## Abstract

T cells have to detect rare high-affinity ‘foreign’ peptide MHC (pMHC) ligands among abundant low-affinity ‘self’-peptide MHC ligands. It remains unclear how this remarkable discrimination is achieved. Kinetic proofreading mechanisms can provide the required specificity but only at the expense of much reduced sensitivity. A number of recent observations suggest that pMHC engagement of T cell receptors (TCRs) induces changes such as clustering and/or conformational alterations that enhance subsequent rebinding. We show that inclusion of induced rebinding to the same pMHC in kinetic proofreading models enhances the sensitivity of TCR recognition while retaining specificity. Moreover, induced rebinding is able to reproduce the striking, and hitherto unexplained, 2D membrane-binding properties recently reported for the TCR.

## Highlights

• We propose that pMHC binding to TCR can increase (induce) pMHC rebinding.
• Published studies are consistent with an induced rebinding model.
• Induced rebinding improves the ability of T cells to discriminate antigens.
• Induced rebinding relates 3D to 2D TCR–pMHC binding parameters.

## The challenge of antigen discrimination

Activation of the adaptive immune response by T cells requires high-affinity engagement of T cell antigen receptors (TCRs) to foreign (e.g., pathogen-derived) peptides bound to MHCs (pMHCs) on antigen-presenting or target cells. A unique feature of TCR recognition is that as a result of positive selection in the thymus, TCRs already bind with somewhat lower affinity to self-pMHCs that are present in high abundance on the surface of the same cells [1,2]. The mechanism by which T cells ignore surfaces expressing only self-pMHC but are activated in response to these same surfaces when they also express even minute quantities of foreign pMHC, a process termed antigen discrimination, remains elusive.

The simplest model relating TCR–pMHC binding to the T cell response is the occupancy model, which postulates that it the total number of TCRs that are engaged determines whether a T cell is activated. This model provides poor discrimination because high occupancy can be achieved for a low-affinity pMHC simply by increasing its concentration. Furthermore, occupancy models have been definitively ruled out by experiments showing that increasing pMHC concentration does not compensate for low affinity [3,4]. Thus, a high concentration of a low-affinity pMHC cannot activate T cells, whereas a pMHC whose affinity is only threefold higher can activate T cells even when presented at extremely low concentrations [3].

**Figure 1:**
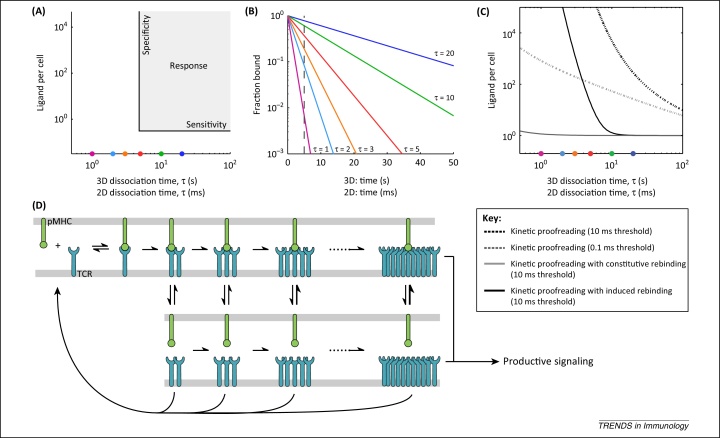
Induced rebinding of T cell receptors improves antigen discrimination. **(A)** Schematic illustrating that T cells need to exhibit a response only to ligands with a dissociation time (*τ* = 1/*k*_off_) above a threshold (vertical line) even when only a single ligand is displayed (horizontal line) while not responding to ligands below the threshold even when displayed in large numbers. **(B)** The fraction of pMHCs that remain bound to the TCR over time for six pMHCs for the dissociation times indicated in units of s (for 3D) or ms (for 2D). Increasing the threshold binding time (vertical line) improves specificity because these lines diverge, but at the cost of sensitivity, because in all cases the fraction of pMHC that remains bound decreases exponentially. **(C)** Direct calculation of the response curves (analogous to panel A) for the standard kinetic proofreading model and kinetic proofreading with constitutive or induced rebinding for the threshold time indicated. **(D)** Schematic of kinetic proofreading with induced rebinding. Note that both 3D and reduced 2D dissociation times are shown (top panels). Coloured circles in panels A and C correspond to the six pMHCs indicated in panel B. Details of the model formulation, calculation, and parameter values can be found in Box 1 and in the supplementary material online.

Recent studies suggest that it is the dissociation time for a TCR–pMHC interaction that is the best predictor of T cell activation [4–7]. The ideal relationship between specificity and sensitivity is illustrated in Figure 1A. Triggering would only be seen in response to a pMHC with a TCR–pMHC dissociation time longer than a given threshold time (vertical line), but is observed at a density of as little as one ligand per cell (horizontal line). Ideally, pMHC ligands with dissociation times below the threshold should not induce a response, even when their concentration is dramatically increased.

T cells are able to respond rapidly to very low levels of agonist pMHC, indicating that they are sensitive to a small number of productive TCR–pMHC interactions. The implications of this are illustrated in Figure 1B, which plots the fraction of pMHCs that remain bound to TCR over time for a wide range of different dissociation times. This reveals that because of the stochastic nature of biochemical interactions, some pMHCs will remain bound to the TCR for the required threshold time among all TCR–pMHC interactions, despite the wide range in dissociation times. From the perspective of the T cell, there will be apparently identical binding events (i.e., lasting similar durations) for pMHC ligands that have different dissociation times. Thus, T cells would be unable to discriminate between abundant pMHCs with short dissociation times and rare foreign pMHCs with long dissociation times.

How do T cells discriminate between pMHC ligands with differences in dissociation time? McKeithan proposed that TCRs use a kinetic proofreading mechanism [8]. This model postulates that each TCR needs to interact with pMHC for a minimum or threshold time, which is longer than the dissociation time, for signalling to occur. Mechanistically, this is achieved by a requirement for a sequence of biochemical modifications before the engaged TCR produces a productive signal, with unbinding of pMHC at any point reverting the TCR to its basal unmodified state. Why should an increase in the required engagement time improve discrimination? This can be appreciated by looking at the fraction of pMHCs that remain bound to the TCR for pMHCs with different dissociation times (Figure 1B); the bound fractions rapidly diverge with time. As a result, an increase in the threshold binding time magnifies small differences in dissociation times. For example, the fraction of pMHC remaining bound for longer than the threshold (vertical line) is 55-fold greater for a pMHC ligand with a dissociation time of 5 s than for one with a dissociation time of 1 s (0.368 vs 0.00674). If the threshold time is increased just twofold, the fraction of pMHCs remaining bound is ∼3000-fold greater for a pMHC with a dissociation time of 5 s than for one with a dissociation time of 1 s (0.14 vs 4.5 × 10^−5^). Thus, specificity can be dramatically improved simply by increasing the time that TCRs need to remain engaged to pMHCs before productive signalling.

However, although the kinetic proofreading mechanism can greatly increase specificity, it does so at the cost of a large reduction in sensitivity. This is because as the threshold time increases, the proportion of all pMHCs that remain bound decreases exponentially (Figure 1B). Direct calculation of a sensitivity–specificity plot for the kinetic proofreading model illustrates this point (Figure 1C). Increasing the threshold improves specificity (note how the large threshold plot is more vertical), but this is at the expense of a large reduction in sensitivity. For example, a pMHC ligand with a dissociation time of 10 s requires < 100 ligands with a smaller threshold, but more than 10 000 ligands with a larger threshold (Figure 1C). This means that very high surface densities of pMHCs (or very long contact periods) are required for productive TCR–pMHC engagement.

In recognition of these shortcomings, there have been a number of proposals to modify the basic kinetic proofreading model to improve antigen discrimination, typically by including feedback processes [3,8–13]. Here we propose a novel modification, motivated by recent experimental observations, in which initial pMHC engagement of TCR induces changes, such as TCR clustering and/or conformational alterations, that greatly enhance rebinding to the same pMHC. We show that inclusion of an induced rebinding mechanism in a kinetic proofreading model restores sensitivity without loss of specificity.

## Extending the kinetic proofreading model by induced rebinding

Several lines of evidence suggest that TCR engagement by pMHC can enhance subsequent binding to pMHC. Fahmy *et al.* observed that activation of T cells enhanced the binding of soluble pMHC dimers to the activated cells [14]. More recently, Zarnitsyna *et al.* demonstrated that pMHC binding as measured using an adhesion assay was enhanced in repeat determinations, a phenomenon they referred to as memory [15]. Finally, TCRs aggregate into nanoscale (10–100 nm) clusters at the cell surface, and this clustering is enhanced by TCR triggering [16–21]. This is relevant because TCR clustering would be expected to enhance pMHC rebinding by increasing the TCR surface density. There are conflicting data concerning the extent to which TCRs form nanoclusters in the resting state [22,23]. One attempt to reconcile these data postulates that TCRs are primarily monomeric in the resting state but are easily triggered under the experimental conditions used for imaging [24].

## Induced rebinding improves antigen discrimination

To investigate the possible effects of induced rebinding on antigen discrimination, we modified the standard kinetic proofreading model to incorporate the formation of a TCR cluster with enhanced binding to pMHC (Figure 1D). We describe the model on the basis that induced rebinding is mediated by TCR clustering for clarity, but emphasise that the model can represent any mechanism of induced rebinding. In this model, the initial binding of pMHC to TCR produces a TCR–pMHC complex that begins to undergo a series of modifications that include TCR clustering. One consequence of this is that when the pMHC dissociates from an intermediate state, it is likely to rebind, and the rate of pMHC rebinding increases as the cluster grows. A productive signal is only transduced by the final mature TCR cluster state. Importantly, the time required to induce a mature cluster is longer than typical pMHC dwell times, but achievement (and maintenance) of this state is greatly enhanced if the pMHC is highly likely to rebind to the cluster each time it dissociates. This model produces a dramatic improvement in discrimination over the kinetic proofreading model, with enhanced sensitivity and specificity (Figure 1C and Box 1; see the supplementary material online for details on model formulation and calculation).

As in the basic kinetic proofreading model, the improvement in specificity is a result of a threshold time that is greater than the typical pMHC dissociation time. Why then does the model not suffer from reduced sensitivity? The key to restoring sensitivity without reducing specificity is that pMHCs that bind for longer selectively increase the rebinding rate. Thus, pMHC unbinding is more likely to be followed by immediate rebinding, increasing the chances the TCR complex will reach and maintain the final productive signalling state. This direct positive feedback helps to restore the sensitivity lost by increasing the threshold time.

An important feature of this model is that the rebinding rate is induced (or increased). If the rebinding rate is constitutively large, as may be the case for pre-existing TCR clusters, it will reduce specificity, because a large rebinding rate from the outset will allow even pMHC with small dissociation times to productively signal (Figure 1C). Further assumptions of the model are discussed in Box 2.

## Induced rebinding modulates the 2D membrane TCR–pMHC binding kinetics

Binding properties between TCRs and pMHCs are typically measured with at least one protein in solution. It may be more appropriate to measure TCR–pMHC binding properties with both TCR and pMHC attached to membranes, as in their native state. Comparison of 3D solution and 2D membrane measurements can elucidate the role of factors such as membrane alignment and mechanical forces in modulating the TCR–pMHC interaction. However, measurement of 2D membrane binding parameters is technically challenging and the relationship between 3D and 2D TCR–pMHC binding parameters remains controversial.

Although there is generally good correlation between 3D binding parameters and functional T cell responses [4,7,25,26], there have been reports of unexpectedly poor correlations between 3D and 2D binding parameters [27–29]. In work by Zhu and colleagues, micropipettes were used to manipulate a live T cell and a pMHC-coupled red blood cell for an adhesion frequency assay [27]. In this assay, the two cells were brought into contact for a defined period of time before being pulled apart to determine if adhesion (and hence pMHC binding) had taken place. This was repeated many times for different time periods to produce a plot of the adhesion frequency over time. This was then used to fit a one-to-one binding model to determine the 2D on-rate and off-rate. After studying a panel of six pMHC ligands, the authors reported that the fitted 2D off-rates correlated inversely with the 3D off-rates. Even more surprisingly, whereas the 3D on-rates did not vary, they measured a 1000-fold variation in the fitted 2D on-rate. This unexpected relationship between 2D and 3D measurements remains unexplained.

**Figure 2:**
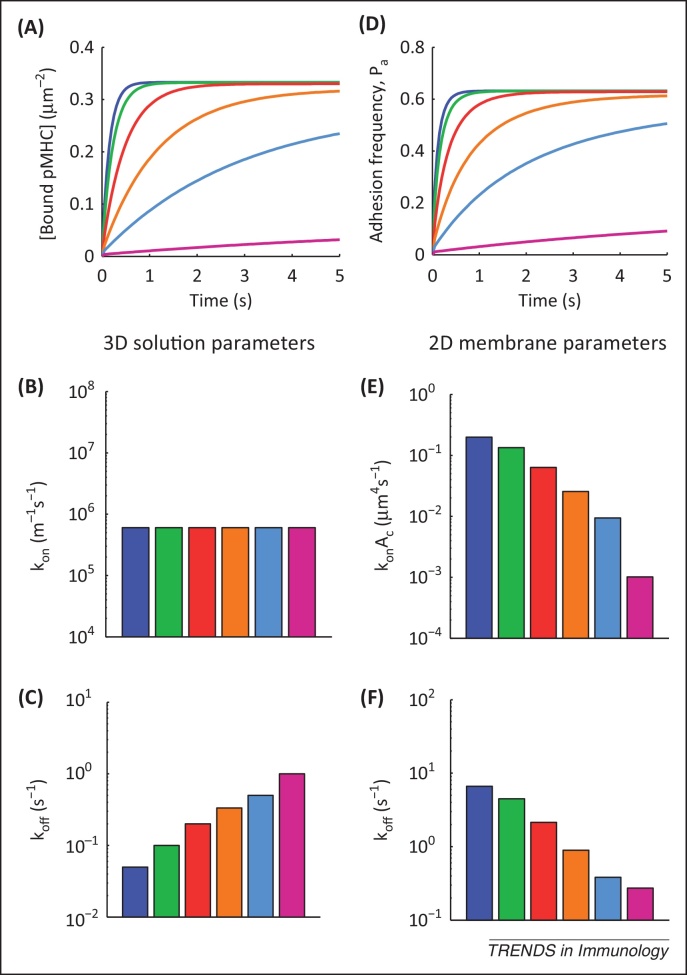
Induced rebinding modulates the fitted 2D TCR–pMHC binding parameters in adhesion frequency assays. In adhesion frequency assays, a T cell and a pMHC-bearing cell are brought into contact for a specific period of time before adhesion (i.e., pMHC binding) is measured. We used the induced rebinding model to simulate the adhesion frequency assay by **(A)** calculating the concentration of bound pMHC over time for six pMHC ligands with **(B,C)** the 3D solution binding parameters indicated. **(D)** The adhesion frequency (or probability of adhesion) is calculated for these six pMHC ligands based on the concentration of bound pMHC. **(E,F)** A fitting procedure is implemented to determine the fitted 2D membrane-binding parameters. Induced rebinding introduces large variance in the fitted 2D on-rates (despite no difference in the 3D on-rates) and produces negative correlation between the 3D and fitted 2D off-rates, as experimentally observed. See the supplementary material online for fitting details.

We hypothesised that induced rebinding could account for these 2D binding parameters. To investigate this we used the induced rebinding model to calculate the concentration of bound pMHC over time (Figure 2A). We used six pMHC ligands with identical 3D on-rates and 3D off-rates that varied 20-fold (Figure 2B,C). Using the concentration of bound pMHC, we calculated the probability that adhesion would occur if the cells were pulled apart (Figure 2D) and determined the fitted 2D membrane parameters from these simulated data according to the procedure used by Zhu and colleagues [27] (Figure 2E,F; see the supplementary material online for details). Strikingly, we found that induced rebinding introduced a very large variance in the fitted 2D on-rate, even though there was no difference in the 3D on-rates (Figure 2B,E). Furthermore, the fitted 2D off-rates were inversely correlated to the 3D off-rates (Figure 2C,F). Thus, induced rebinding can explain the unexpected relationship between 3D and 2D binding properties reported by Zhu and colleagues [27].

The mechanism underlying the relationship between the 3D and fitted 2D binding parameters predicted by the model is based on domination of the adhesion frequency by induced rebinding. The fitted 2D off-rates are determined by how quickly the adhesion frequency assay reaches a steady state, and this is reached faster (larger fitted 2D off-rate) for pMHCs with a smaller 3D off-rate because these ligands induce maximal rebinding (and hence maximal adhesion) more quickly. This accounts for the negative correlation between 3D and fitted 2D off-rates. The wide range for fitted 2D on-rates is explained on the basis of differentially induced rebinding based on the 3D off-rate and on differences in the fitted 2D off-rate. This accounts for the negative correlation between the 3D dissociation time and the fitted 2D on-rates. Importantly, constitutive or negligible rebinding could not explain these relationships (data not shown). Thus, our model implies that the physiological fitted 2D binding parameters determined by Zhu and colleagues [27] are the result of processes that induce rebinding, such as TCR clustering, conformational changes, and/or membrane alignment.

Correlations between the fitted 2D binding kinetics and functional T cell responses, as measured by T cell proliferation and TCR downregulation, produced a puzzling result [27]: although the fitted 2D on-rate showed a positive correlation with pMHC potency, so did the fitted 2D off-rate, suggesting that pMHC ligands with larger 2D off-rates are more potent T cell stimulators. The induced rebinding model provides a rationale for this observation by suggesting that the 3D off-rate determines the extent of TCR triggering, which in turn determines the extent of induced rebinding and therefore the fitted 2D binding parameters (as described above). It follows that correlations between the functional T cell response and the fitted 2D binding properties are a direct result of the 3D binding parameters. An induced rebinding mechanism is thus able to reconcile previous apparently contradictory reports on the dependence of TCR triggering on 2D and 3D binding properties.

## Comparison with other models of antigen discrimination

Several modifications have been proposed for the basic kinetic proofreading model to improve the trade-off between specificity and sensitivity. McKeithan proposed that the final productive TCR–pMHC complex has a much longer dissociation time [8]. However, such large changes in TCR–pMHC dissociation time have not been observed. Cooperativity between TCRs in the form of negative feedback produced by TCRs in intermediate signalling states improves specificity, but again at a cost to sensitivity [9,10]. Extending the negative feedback models by including a positive feedback partially restores the loss in sensitivity [3,9–11]. A key drawback of these feedback models, which are mediated by intracellular signalling pathways, is that feedback effects would be dispersed between TCRs engaging different pMHCs, perhaps even on different cells. As a result, T cell responses to specific pMHCs would be disturbed by cross-talk with other pMHCs. Thus, a response to one cell bearing a foreign pMHC could be suppressed by simultaneous interaction with a second cell bearing only self-pMHC. By contrast, our induced rebinding model is free of such cross-talk. A previous model of TCR–pMHC rebinding failed to observe improved antigen discrimination based on the dissociation time, because individual TCRs in a cluster acted independently in that model [12].

## Mechanism of induced rebinding

Further studies are required to explore the mechanism of enhanced rebinding (Box 2). As noted above, there is reasonable evidence of TCR clustering, but the clustering mechanism is not understood. One possibility is that it is mediated by proteins recruited to the TCR cytoplasmic domains following triggering. For example, the CD4 and CD8 coreceptors are recruited to triggered TCR/CD3 via Lck and ZAP-70 recruited to the cytoplasmic domains [30]. The large number of TCR/CD3 cytoplasmic domains would facilitate rapid extensive clustering. Phosphorylation of TCR/CD3 cytoplasmic domains also leads to their dissociation from the plasma membrane [31,32], which may enhance clustering by enhancing mobility and reducing steric inhibition [33]. Quantitative information about clustering is also lacking. Although it is not known how rapidly TCRs form nanoclusters, on the basis of reasonable estimates for diffusion coefficients and cluster size, they could form within 0.01 s (Figure S3 in the supporting material online). Rebinding could be enhanced by other mechanisms, such as reorientation of the TCR to make it more accessible to pMHC. Coreceptor recruitment to the TCR–CD3 complex would be expected to facilitate rebinding, although this appears to be a slower process [34]. Finally, initial signalling could induce cytoskeletally driven processes that improve proximity and alignment between the membranes [35,36].

## Concluding remarks

The basic kinetic proofreading model can provide the specificity required for TCR recognition, but this is at the expense of a loss of sensitivity. Modifications of the model to include a feedback mechanism can enhance specificity and sensitivity, but this is at the expense of cross-talk between different pMHCs, which is potentially problematic. The induced rebinding model described here can optimise specificity and sensitivity without introducing cross-talk between pMHCs. The model is supported by several lines of evidence, including observation of TCR clusters and enhanced TCR binding, and it provides an explanation for the intriguing, and hitherto unexplained, relationship between the 3D and fitted 2D binding parameters.

To validate (or refute) the model, experiments are needed to directly observe induced rebinding and to show its impact on antigen discrimination. Direct observation of induced rebinding is challenging because it requires monitoring of the dissociation time for single TCR–pMHC interactions at 2D interfaces in live cells with high temporal resolution. Recent studies suggest that this may be feasible [27,28]. The model predicts that induced rebinding may depend on active processes, such as signalling-induced TCR clustering and/or cytoskeleton-induced membrane alignment, and therefore blocking these should reduce the effective TCR–pMHC dissociation time. We note that such experiments need to perturb the induced rebinding process and therefore cannot be conducted on a steady-state mature synapse. Once mechanism(s) of induced rebinding are known, their contribution to antigen discrimination can be determined by examining the T cell response to panels of pMHC ligands, as in previous work [3,4], in the presence and absence of induced rebinding. We hope that future experiments will elucidate the precise mechanism(s) of induced rebinding and their contribution to antigen discrimination.

## Box 1 Mathematical model.

To model kinetic proofreading with induced rebinding, we modified the standard kinetic proofreading model to allow for pMHC rebinding. To do this, we assume that on pMHC dissociation, intermediate states are formed in which the pMHC remains in spatial proximity to TCR(s), whose modification state persists despite being unbound from pMHC (Figure 1D, bottom row). In these intermediate states, three possible reactions can take place: pMHC can immediately rebind, pMHC can diffuse away and/or the TCR modifications can be reversed, or TCR modifications continue (Box 2). Induced rebinding is included by increasing the rebinding rate with each modification (step) in the proofreading scheme.

The biochemical interactions (e.g., TCR–pMHC binding interactions and TCR modifications or signalling), their mathematical formulation, and the numerical solutions of the mathematical model are determined as in previous studies [3,8,12,13] (see the supplementary information online). As in previous studies, we used deterministic ordinary differential equations (ODEs) to determine the mean number of activated TCRs per T cell, and a T cell response is said to have taken place when this number exceeds one. Alternatives to deterministic calculations are stochastic simulations in which reactions are based on probability distributions defined by reactant concentrations and reaction rate constants. The probabilistic nature of stochastic simulations means that each simulation typically produces a different result, such as the number of TCRs activated. The simulation can be repeated many times to produce the distribution of activated TCRs for a population of T cells, from which the mean can be calculated. Our goal is to determine the mean, so we directly calculated this quantity using ODEs. A recent study by Francois *et al.* revealed good correspondence between deterministic and stochastic simulations of kinetic proofreading [13].

## Box 2 Mathematical model assumptions

**Rate of rebinding**. The precise mechanism of induced rebinding is unknown and therefore the rate of rebinding is not known. Without this information, we assumed a non-linear 10 000-fold increase in the rebinding rate from an initial rate of 10^3^ s^−1^. This initial rate is approximated based on a single TCR and pMHC confined to an area of approximately 100 nm^2^, and the increase reflects TCR clustering, conformational change, and/or membrane proximity (see the main text for a discussion of induced rebinding mechanisms). Experimental work is required to directly measure the rebinding rate and how this rate is modulated on signalling initiated by pMHC binding to the TCR. As this information becomes available, it can be used to inform the interplay between rebinding and, for example, clustering in mathematical models [12,37–40].

**Signalling threshold**. The threshold time for interaction between a pMHC and a TCR (or a TCR cluster) before a productive downstream signal is initiated is unknown at present. Experiments have shown that tyrosine phosphorylation occurs within a few seconds of pMHC binding [41] and an increase in the adhesion frequency is observed in < 1 s [27]. Assuming that the increase in adhesion frequency is related to an increase in induced rebinding (as we have argued), this suggests that the threshold time must be shorter than these time scales, so we assumed a threshold of 10 ms. The precise value does not alter our conclusions, but does determine the time scale on which rebinding is induced and consequently the time scale of antigen discrimination.

**Relationship between 3D and 2D binding parameters**. All mathematical models of TCR–pMHC interactions require 2D binding parameters. Therefore, we converted 3D binding parameters to 2D binding parameters using multiplicative constants. The precise values of these constants are not important for any of our conclusions, but they do impact on the time scales in our calculations. For example, the assumption of a threshold time of 10 ms above implies that T cells discriminate antigens with 2D dissociation times on this scale (or equivalently with 2D off-rates of ∼100 s^−1^). This means that for T cell discrimination of antigens with a 3D dissociation time of ∼10 s, the conversion between 3D and 2D off-rates must rely on a multiplicative constant of approximately 1000.

The values of these multiplicative constants are unknown and it is also unknown whether conversion between 2D and 3D binding parameters requires a single constant value or exhibits a more complex relationship [42]. Experiments to determine 2D membrane TCR–pMHC interactions are challenging [27,28] and further work is required to determine how the fitted 2D rate constants depend on TCR-induced signalling and ultimately on their 3D counterparts.

**Proofreading independent of pMHC binding**. We assumed that TCR modifications (proofreading) continue to occur during brief periods of pMHC dissociation (Figure 1D, bottom row). This captures the intuition that during very brief dissociation events, the TCR remains in a membrane environment that favours phosphorylation (e.g., segregation of CD45). This is consistent with the kinetic segregation model of TCR triggering [43]. Our conclusions are unchanged if proofreading is dependent on pMHC binding.
