# Supplementary material for: An induced rebinding model of antigen discrimination
Source: Trends Immunol. 2014 Apr;35(4):153–8. doi: 10.1016/j.it.2014.02.002 (PMC3989030; doi:10.1016/j.it.2014.02.002)
Supplement: Supplementary file 1 [file mmc1.pdf]

Supplementary Information:
An induced rebinding model of antigen discrimination

Omer Dushek[†,‡,¶], P. Anton van der Merwe[†,¶]

[†]Sir William Dunn School of Pathology, University of Oxford
[‡]Wolfson Centre for Mathematical Biology, University of Oxford
[¶]Corresponding author

Mathematical model for kinetic proofreading with induced rebinding

As discussed in the main text, there are multiple mechanisms that can generate induced rebinding, including TCR clustering, conformational change, and enhanced membrane proximity/alignment. For clarity, we present the mathematical model using the semantics that induced rebinding is mediated by induced clustering.

The system of ordinary-differential-equations (ODEs) corresponding to the reaction scheme shown in Fig. S1 (analogous to Fig. 1D except with all chemical states and parameters labelled) is as follows:

$$\frac{\partial P}{\partial t} = -k_{\text{on}} P T + k_{\text{off}} C_0 + \lambda \sum_{i=1}^{N} C_i^* \tag{1}$$

$$\frac{\partial T}{\partial t} = -k_{\text{on}} P T + k_{\text{off}} C_0 + \lambda \sum_{i=1}^{N} C_i^* \tag{2}$$

$$\frac{\partial C_0}{\partial t} = k_{\text{on}} P T - (k_{\text{off}} + k_{\text{p}}) C_0 \tag{3}$$

$$\frac{\partial C_1}{\partial t} = k_{\text{p}} C_0 - (k_{\text{off}} + k_{\text{p}}) C_1 + \rho_i C_1^*, \tag{4}$$

$$\frac{\partial C_1^*}{\partial t} = k_{\text{off}} C_1 - (\rho_i + \lambda + k_{\text{p}}) C_1^*, \tag{5}$$

$$\frac{\partial C_i}{\partial t} = k_{\text{p}} C_{i-1} + \rho_i C_i^* - (k_{\text{off}} + k_{\text{p}}) C_i \quad i = 1..N-1 \tag{6}$$

$$\frac{\partial C_i^*}{\partial t} = k_{\text{p}} C_{i-1}^* + k_{\text{off}} C_i - (\rho_i + \lambda + k_{\text{p}}) C_i^*, \ i = 1..N-1 \tag{7}$$

$$\frac{\partial C_N}{\partial t} = k_{\text{p}} C_{N-1} + \rho_N C_N^* - k_{\text{off}} C_N \tag{8}$$

$$\frac{\partial C_N^*}{\partial t} = k_{\text{p}} C_{N-1}^* + k_{\text{off}} C_N - (\rho_N + \lambda) C_N^* \tag{9}$$

where P is the concentration of free pMHC (in units of μm^{-2}), T is the concentration of free TCR (in units of μm^{-2}), C_i is the concentration of bound TCR clusters with i modifications ($i = 1..N$ where N is the maximum number of modifications), and C_i^* is the concentration of unbound TCR clusters with i modifications. The model parameters include: k_{on} as the two-dimensional on-rate (in units of $\mu\text{m}^2\text{s}^{-1}$), k_{off} as the two-dimensional off-rate

(in units of s^{-1}), k_p as the modification rate (in units of s^{-1}), ρ_i as the rebinding rate of pMHC to a TCR cluster with i modifications, and λ as the signaling decay rate (in units of s^{-1}), which determines the lifetime of an unbound modified TCR cluster (i.e. increasing λ reduces the time that modified TCRs maintains high rebinding rates). The model accounts for induced rebinding by assuming that the rebinding rate (ρ_i) increases as the modification (signaling) state of the TCR cluster increases from $i = 1$ to $i = N$. The precise values of ρ used are discussed below.

Table 1: Summary of parameter definitions.

Parameter	Description	Units
T	Free TCR concentration	μm^{-2}
P	Free pMHC concentration	μm^{-2}
k_{on}	Two-dimensional on-rate	$\mu m^{-2} s^{-1}$
k_{off}	Two-dimensional off-rate	s^{-1}
k_p	TCR cluster modification rate	s^{-1}
ρ_i	pMHC rebinding rate to TCR in state i	s^{-1}
λ	modified TCR decay rate	s^{-1}

As discussed in the main text, the mathematical model we formulate is phenomenological in nature because the underlying mechanisms of many of these experimentally observed processes are presently unknown. Furthermore, quantitative details on how these mechanisms increase the rebinding rate is missing (e.g. it is unknown how the concentration of TCR changes over time as a TCR cluster is induced). As quantitative experimental evidence becomes available the model can be modified to capture these mechanistic details.

Calculations for antigen discrimination (Fig. 1)

All calculations are initialized with all TCR and pMHC unbound. The system of ODEs are integrated for 120 seconds and the number of productively signaling TCRs ($A(C_N + C_N^*)$, where A is the T cell surface area taken to be $4\pi 5^2$ μm^2) is recorded. A T cell response is said to have taken place only if the number of productively signaling TCRs at 120 s is greater than 1 (i.e. $A(C_N + C_N^*) > 1$). Calculations for longer time periods do not alter the results in Fig. 1C.

The free TCR concentration is $T = 100$ μm^{-2} and the pMHC concentration is varied (the number of ligands per cell is calculated as $P \times A$). We have assumed that all pMHC have identical 3D on-rates of 0.6×10^6 $M^{-1}s^{-1}$ and the indicated 3D off-rates (or dissociation times, $\tau = 1/k_{off}$). The mathematical model requires 2D reaction rates and we therefore estimated the 2D on-rate to be $k_{on} = 0.1$ $\mu m^2 s^{-1}$ (using a confinement length of 10 nm) and estimated the 2D off-rate by accelerating the 3D rates by a factor of 10^3. The precise conversion factors are unknown but are not important to the qualitative conclusions.

The total number of modifications was taken to be $N = 25$. The rate of modifications, k_p, was varied to achieve a threshold of 0.1 ms ($k_p = 250000$ s^{-1}) or a threshold of 10 ms ($k_p = 2500$ s^{-1}), as indicated. The rate at which a modified TCR cluster returns to the basal state when pMHC unbinds (λ in our model) is unknown. In the standard kinetic proofreading model this rate is not limiting so that unbinding instantaneously returns TCRs to their basal state. However, we are including fast rebinding timescales and therefore cannot make this assumption. As an approximation, we take $\lambda = 10^4$ s^{-1} which is consistent with the rate of dephosphorylation by abundant phosphatases (1) and is similar to the rate at which membrane proteins diffuse on the nm scale (2).

The relationship between the rebinding rate and the modification state of the TCR we have used is shown in Fig. S2. The initial rate of rebinding (without clustering or conformational change) can be approximated to be 10^3 s^{-1}, which can be arrived at by taking the on-rate over the area of a single TCR (0.1 $\mu m^2 s^{-1}$ / 0.01^2 μm^2) (1). How this rebinding rate is modified by clustering, conformational change, and/or membrane alignment is unknown and we have assumed a non-linear increase with a maximum rebinding rate of 10^7 s^{-1}. Substituting the non-linear increase in rebinding with a constant or a linear increase reduced the capacity to discriminate antigens.

Calculations for two-dimensional reaction rates (Fig. 2)

To simulate the adhesion frequency assays used to determine the TCR/pMHC two-dimensional reaction rates (3), we have used a small pMHC concentration of $P = 1/3$ μm^{-2} so that the small contact area used in the assay ($A = 3$ μm^{-2}) will produce on average only a single pMHC. All other concentrations and reaction parameters are identical to the calculation for antigen discrimination (namely, $T = 100$ μm^{-2}, $k_{\mathrm{p}} = 2500$ s^{-1}, $\lambda = 10^4$ s^{-1}, and ρ as indicated in Fig. S2).

We assume initially ($t = 0$ s) that all pMHC and TCR are free and calculate the concentration of bound pMHC as a function of time ($C = \sum_{i=0}^{N} C_i$, denoted as [Bound pMHC] in Fig. 2A). We use C to determine the adhesion frequency (P_A, probability of finding a pMHC bound) using the following relation (3):

$$P_A = 1 - e^{-C \times A} \tag{10}$$

The adhesion frequency (P_A) is shown in Fig. 2D. We then follow the procedure of Huang et al (3) to fit the following equation to the adhesion frequency to estimate the two-dimensional affinity ($K_A A_C$) and off-rate (k_{off}):

$$P_A = 1 - e^{-n} \tag{11}$$

where n is

$$n = TPK_A A_C (1 - e^{-k_{\mathrm{off}} t}) \tag{12}$$

Using *lsqcurvefit* in Matlab (Mathworks, MA) we fit equation 11 to the simulated adhesion frequency data (Fig. 2D) to obtain the fitted values of $K_A A_C$ and k_{off} and use these values to estimate $k_{\mathrm{on}} A_C$ ($k_{\mathrm{on}} A_C$ = $K_A A_C \times k_{\mathrm{off}}$).

Using this procedure we calculate the adhesion frequency assay for 6 pMHC ligands with the indicated 3D on-rates and off-rates (Fig. 2B,C). We convert these 3D rates into 2D rates required by the mathematical model, as described in the antigen discrimination section above, in order to calculate the concentration of bound pMHC (Fig. 2A), which is used to determine the adhesion frequency (Fig. 2D), which itself is used to determined the fitted 2D membrane parameters (Fig. 2E,F). We note that the 2D rate constants that are specified in the mathematical model are intrinsic biophysical constants whereas the 2D fitted membrane parameters determined by the adhesion frequency assay are physiological (apparent) fitted parameters that depend on, for example, signaling and clustering.

This calculation shows that induced rebinding is a mechanism that can introduce large variation in the fitted 2D on-rate despite constant 3D on-rates (Fig. 2B vs. Fig. 2E) and introduces an inverse correlation between the fitted 2D off-rate and the 3D off-rates (Fig. 2C vs. Fig. 2F). What is the mechanism underlying these intriguing relationships? The fitted 2D membrane off-rate is approximately determined by how quickly the adhesion frequency reaches steady-state (Fig. 2D) and this is reached faster (larger fitted 2D off-rate) for pMHC ligands with small 3D off-rate since it is these ligands that readily reach the final kinetic proofreading step where large rebinding is induced. Moreover, the large differences in the fitted 2D on-rates are a direct result of large differences in the fitted

2D off-rates because the fitted 2D affinities are similiar for all six pMHC ligands tested (i.e. they have similar steady-state adhesion frequencies). In this way, the experimentally determined adhesion frequency may be dominated not by a single pMHC interacting with a single TCR but by a single pMHC interacting with, for example, an induced cluster of TCRs where rebinding is prevalent.

Estimating the timescale of induced clustering

This calculation previously appears in Text S2 of Dushek et al (1) based on the work of DL Weaver (4).

We can obtain an estimate for the time required to form a TCR cluster using the approximate theory of Weaver (4). The model assumes a diffusion-mediated localization to a disc (cluster) on the surface of a sphere which is irreversible. The number of TCR in the cluster (T_{mc}) as a function of time follows

$$T_{\mathrm{mc}}(t) = TA_{\mathrm{mc}} + TA_{\mathrm{s}}(1 - \exp(-t/t_p))$$

where T is the background cell surface TCR concentration, A_{mc} is the TCR cluster area, A_{s} is the cell surface area, and t_p is given by

$$t_p = \frac{r_s^2}{D}\left[\frac{A_{\mathrm{s}}/A_{\mathrm{mc}}}{A_{\mathrm{s}}/A_{\mathrm{mc}} - 1}\ln(A_{\mathrm{s}}/A_{\mathrm{mc}}) - 1\right]$$

where r_s is the cell radius and D is the TCR diffusion coefficient. We obtain an estimate for the length of time required to localize $T^*_{\mathrm{mc}}A_{\mathrm{mc}}$ TCR by solving for t_{f} when $T_{\mathrm{mc}}(t_{\mathrm{f}}) = T^*_{\mathrm{mc}}A_{\mathrm{mc}}$,

$$t_{\mathrm{f}} = t_p \ln\left[\frac{TA_{\mathrm{s}}}{TA_{\mathrm{s}} + TA_{\mathrm{mc}} - T^*_{\mathrm{mc}}A_{\mathrm{mc}}}\right]$$

where T^*_{mc} is the final clustered TCR concentration.

In Fig. S3 we show heat maps of t_{f} as a function of the diffusion coefficient (D) and the TCR cluster area (A_{mc}) for a T^*_{mc} to T ratio of $T^*_{\mathrm{mc}}/T = 2$ (Fig. S3A), $T^*_{\mathrm{mc}}/T = 5$ (Fig. S3B), and $T^*_{\mathrm{mc}}/T = 10$ (Fig. S3C). The parameters used are as follow: $r_s = 5\ \mu$m, $A_{\mathrm{s}} = 4\pi r_s^2$, and $T = 30000/As\ \mu\mathrm{m}^{-2}$ (assuming 30,000 TCR per T cell).

References

1. Dushek O, Das R, Coombs D (2009) A role for rebinding in rapid and reliable T cell responses to antigen. *PLoS computational biology* 5:e1000578.

2. Dushek O, van der Merwe PA, Shahrezaei V (2011) Ultrasensitivity in multisite phosphorylation of membrane-anchored proteins. *Biophysical journal* 100:1189–97.

3. Huang J, et al. (2010) The kinetics of two-dimensional TCR and pMHC interactions determine T-cell responsiveness. *Nature* 464:932–6.

4. Weaver D (1983) Diffusion-mediated localization on membrane surfaces. *Biophysical journal* 41:81–86.

Supplementary Figures

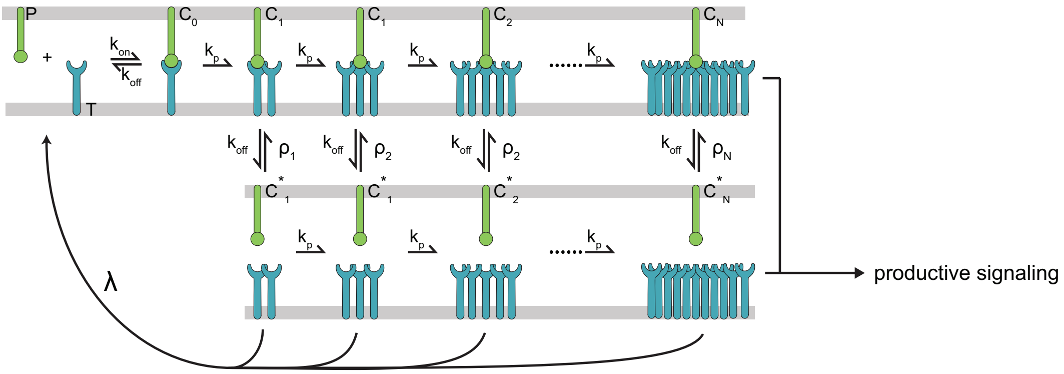

Fig S1: Schematic of the induced rebinding model, as shown in Fig. 1D, but with all chemical states and reaction parameters labelled.

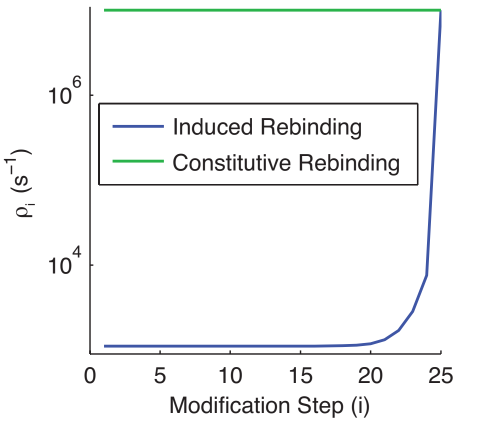

Fig S2: The rate of rebinding (ρ) over the modification steps (i) for the kinetic proofreading with induced and constitutive rebinding.

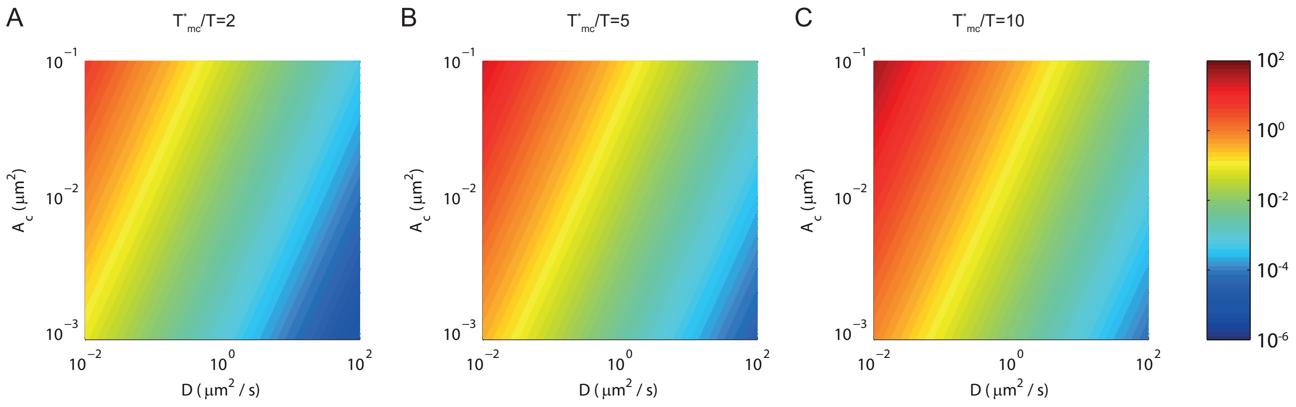

Fig S3: Estimated time to form a TCR cluster. Shown are heat maps of the estimated time to form a TCR cluster (in units of seconds) as a function of the diffusion coefficient (x-axis) and the TCR cluster area (y-axis) for the case when the final TCR cluster concentration is A) 2, B) 5, or C) 10-fold larger than the background TCR concentration. With a cluster area of 10^{-2} μm^2 and a local diffusion coefficient of 1 $\mu m^2 s^{-1}$, a TCR cluster can be induced on a millisecond timescale. See Supplementary Information for details.

Fig 1:

Fig 2:
